# Developing a novel functional disc emulator to investigate the nucleus pulposus replacement

**DOI:** 10.1007/s10856-021-06492-z

**Published:** 2021-03-10

**Authors:** Hassan M. Raheem, Skip E. Rochefort, Brian K. Bay

**Affiliations:** 1 Faculty of Engineering, Mechanical Department, University of Kufa, Najaf, Iraq; 2 Ministry of Oil, Midland Refineries Company, Karbala Refinery, Karbala, Iraq; 3 School of Chemical, Biological, and Environmental Engineering, Oregon State University, Corvallis, OR USA; 4 School of Mechanical, Industrial, and Manufacturing Engineering, Oregon State University, Corvallis, OR USA

## Abstract

We have developed a simple, inexpensive and innovative device for reproducing the global mechanical behavior of spinal motion segments and the local mechanical environment experienced by lumbar intervertebral discs. The device has several broad functions: (1) exploration of the basic mechanics underlying this complex skeletal system, (2) connecting changes in tissue characteristics with overall motion segment function, and (3) evaluation of strategies for repair and replacement of disc components. This “disc emulator” consists of three main parts: (1) an artificial annulus fibrosus (AAF), made out of silicone, with lumbar disc geometry and adjustable material properties, (2) a hydrogel nucleus pulposus (NP) also with lumbar disc geometry and adjustable material properties, and (3) simulated vertebral bodies 3D printed with trabecular bone simulated by a rigid polymer (Acrylonitrile Butadiene Styrene, ABS) and end plates crafted from a compliant polymer (Thermoplastic Polyurethane, TPU). Mechanical compression experiments have been conducted using the disc emulator under similar protocols to published studies of human cadaver samples. Bulging of the artificial annulus fibrosus was examined under axial compression loads using digital image correlation (DIC), and results show close agreement. We see this approach of using anatomical geometry and multiple adjustable components as a useful means of creating accurate local stress/strain environments for preliminary material evaluation, without the variability and difficulty inherent indirect testing of cadaveric materials.

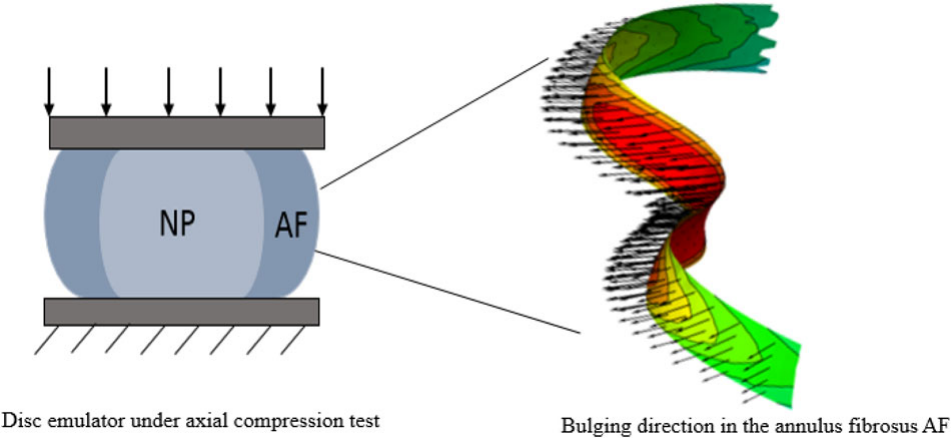

## Introduction

Intervertebral discs (IVD) are crucial components of the spinal column as they provide both flexibility and support of the very large compression, bending, and torsional loads that occur during daily activities. Unfortunately, IVD is also susceptible to degradation and damage. Low back pain is the third most common reason for surgery in the United States [1]. Over a million patients in the USA annually undergo surgery to counteract the effects of disc herniation [2]. Understanding the factors leading to disc failure, improving treatment strategies, and developing approaches to prevention are major goals in orthopedic practice.

Overall kinematics of the spine are, to a limited degree, accessible through studies of human subjects, but detailed tissue-level mechanics are not. Researchers have employed a variety of approaches to bridge this gap, with studies generally falling into three broad categories: (1) measurements on human cadaveric material [3, 4], (2) studies of samples from various animal analogues [5], and (3) analysis with finite element or other computational models [6–9].

All these approaches provide valuable information but suffer from typical limitations associated with biomechanics research. Human cadaveric material is generally older, somewhat degenerated already (particularly spines), and inherently variable. Animal models are younger (often inconveniently so) and more consistent, but exhibit differences from human samples in geometry, tissue properties, and cellular composition. Finite element models are simplified representations of very complex biomechanical systems and require input of parameters (material properties, loading conditions) with varying levels of uncertainty.

There are many patents [10–12], and several studies proposed prosthetic disc [13] that replaces the whole intervertebral disc, or they modeled the synthetic nucleus pulposus (NP), i.e., hydrogel [14, 15]. However, the devices that available are not meant to be used in the lab for testing the materials for NP replacement. The devices are usually used in replacing the damaged discs or could be implanted in the spinal column to retrieve the mechanical function of the spinal segment. Furthermore, they are very complicated to be manufactured and they have required highly skilled personnel to deal with and, to some extent, required an invasive intervention to be implemented. There is also a paucity of studies that report a physical model, i.e., an apparatus, which is meant to be used for testing proposed materials for replacing the degenerated NP. Thus, in this study, the disc emulator can be easily reproduced with minimum cost and a moderate knowledge about using 3D printing technologies. These features make the disc emulator unique. It is worthy to mention that the disc emulator was tested without the contribution of lateral joints.

These limitations led us to a new approach. With the advent of additive manufacturing and the ongoing rapid introduction of polymer materials within that framework, it has become feasible to create test systems of complex, customized geometry from materials that mimic biological tissues.

To the best of the authors’ knowledge, there are no studies in the literature that have developed a physical model that reproduces the geometry and mechanical response of a lumbar spinal motion segment in terms of evaluating the materials for the nucleus pulpous replacement. Thus, the goal of this program is to develop and characterize an apparatus that reproduces the anatomy and mechanics of spinal motion segments, but fully from synthetic materials. This initial report focuses on compressive loading as a fundamental aspect of the overall spine mechanical environment [16]. Bending, torsion, and combined loading could be added without changing the overall approach. A more basic goal was development of a compartmentalized but realistic simulation of a motion segment, with the ability to simulate normal and compromised tissue characteristics and assess the mechanical performance of replacement and repair materials.

A spinal motion segment is comprised of two vertebral bodies and the interposed intervertebral disc. The IVD consists of two primary components: the outer annulus fibrosus (AF), and the central NP. Cartilaginous end-plates provide an interface between the IVD and the vertebral bodies. The structure is very complex and tightly integrated, and we are not claiming to have created an analogue of high fidelity. But we are establishing a construct with representative overall anatomic geometry and replaceable/adjustable components that can evolve toward more realistic simulations. For example, the AF component in this study is a simple silicone structure with an overall stiffness adjusted to match existing lumbar spine test data. The actual AF is comprised of fiber-reinforced layers, with considerable variation in layer thickness, curvature, and fiber structure/orientation [17]. As additive manufacturing processes evolve, disc emulator construction will evolve as well.

We first established the overall geometry of the components using available information on the anatomy of the human lumbar spine [18, 19]. We then measured disc emulator compressive response with an empty NP compartment, comparing results with published data from denucleated human lumbar spines, as a means of tuning the AF material properties. Hydrogel NP components were then molded with a broad range of stiffness to assess the influence on overall construct behavior. The complete construct was evaluated in compression and results compared with a broad range of test and simulation results.

Finally, to validate the disc emulator further, bulging of the artificial annulus fibrosis was examined using a non-contact measurement, an optical method using digital image correlation (DIC). The first implementation of using DIC in biomechanics was in the 90 s [20]. Since then, many studies have used DIC for such measurements [21]. The DIC, which is an attractive technique in testing soft and biomechanics materials, allowed the measuring of displacement and calculation of strain for the area of interest in soft and hard samples.

## Materials and methods

### Materials

**Fig. 1:**
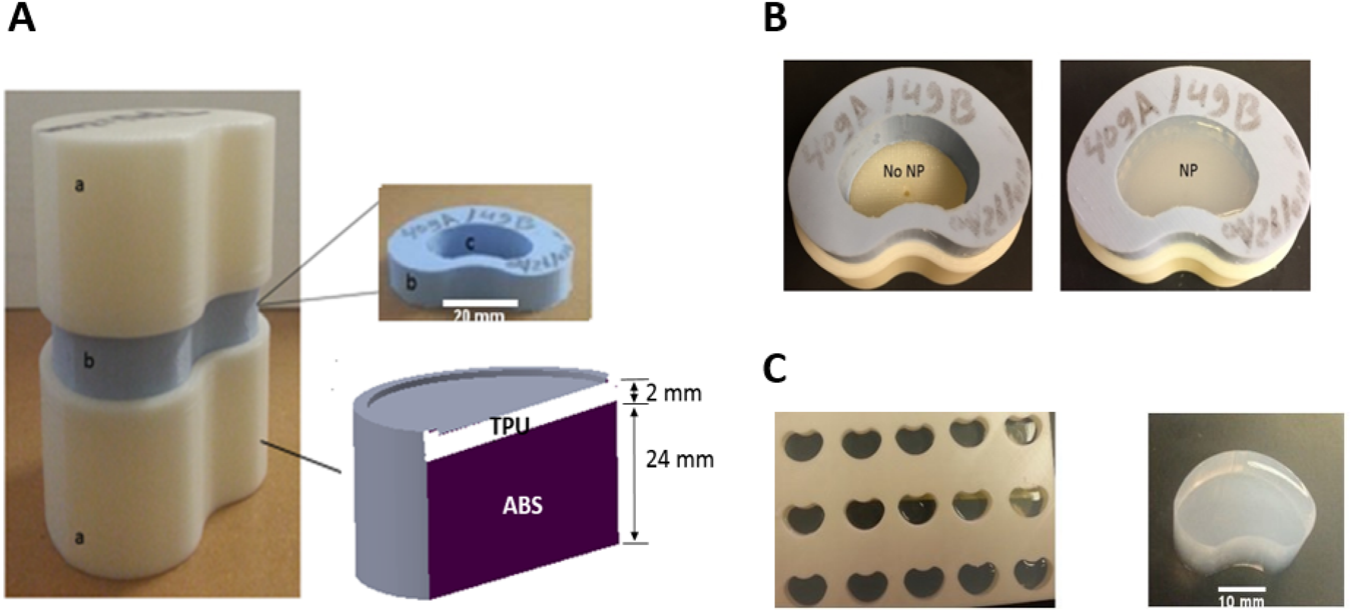
**A** The disc emulator assembly. a Artificial vertebral bodies of ABS plastic with a 2 mm layer at the surface of TPU. b Artificial annulus fibrosus of RTV 630 silicon (c) cavity for nucleus pulposus simulation materials (**B**) annulus fibrosus component denucleated (left) and with a hydrogel nucleus pulposus (right) (**C**) mold for casting hydrogel NP components (left) and an example (right)

The disc emulator, which is illustrated in (Fig. 1A), has three main components: (1) vertebral bodies with the lumbar segment contours, (2) an annulus with suitable height and wall thickness, and (3) a compartment for the introduction of a NP analogue. The vertebrae were 3D printed using Acrylonitrile Butadiene Styrene (ABS) representing the bony components and Thermoplastic Polyurethane (TPU) representing the more compliant endplates (not porous in this initial version).

The artificial annulus component, see (Fig. 1B), was made of silicone (RTV-630, NY, USA), similar to [22]. The stiffness of the silicone can be adjusted by varying the ratio of base and curing agent, with a ratio of 10:1 producing appropriate load response. The geometry of the annulus was matched in overall height, shape, and wall thickness variation to anatomic data. This version is a simple monolithic material without layering or internal fiber reinforcement. The annulus was glued to the vertebral body components with cyanoacrylate to allow pressurization of the NP component when present.

Agarose (IBI Scientific, IA, USA) was used for the NP component as a generic hydrogel analogue [23], as shown in (Fig. 1C). The powder was dissolved in heated deionized water at 90 °C then poured into premade molds matching the interior dimensions of the annulus. Samples were left to cool to room temperature, removed from the mold, and kept in a plastic bag stored in a refrigerator until the day of the test. Formulations of 1.5 and 4 weight % (*wt*. %) were used to create a range of stiffness and strength characteristics. In addition to the molded shapes, disc-shaped samples 4 mm in height and 25 mm in diameter were made for unconfined compression tests.

### Test methods

All tests were conducted on an Instron 5567 material testing system equipped with a 2 kN load cell. Unconfined compression tests of the hydrogel discs (*n* = 5) were conducted at a displacement rate of 1 mm/min until load drop to establish the stiffness characteristics of the two formulations (1.5 and 4.0 *wt*. %). Stress and strain were calculated as engineering values based on initial sample dimensions.

Tests using the disc emulator construct generally followed the methods of [4]. The device was placed between compression platens within the load frame, with the lower vertebral body rigidly fixed and the upper body under moment relief. An initial 50 preconditioning cycles of 3% nominal axial strain were applied, followed by steady axial compression at 15% strain/sec to a total of 15% strain. Overall load/displacement data were recorded.

Initial tests with the NP compartment empty were conducted to assess the overall stiffness of the annulus independent of pressurization effects, with results compared to denucleated human spine data. The two hydrogel formulations were then tested within the NP space to evaluate whether material stiffness or pressurization effects had the greater impact on construct stiffness. Data for the intact construct with the 4 *wt*. % of agarose nucleus was then compared with a range of literature results.

### Digital image correlation (DIC)

**Fig. 2:**
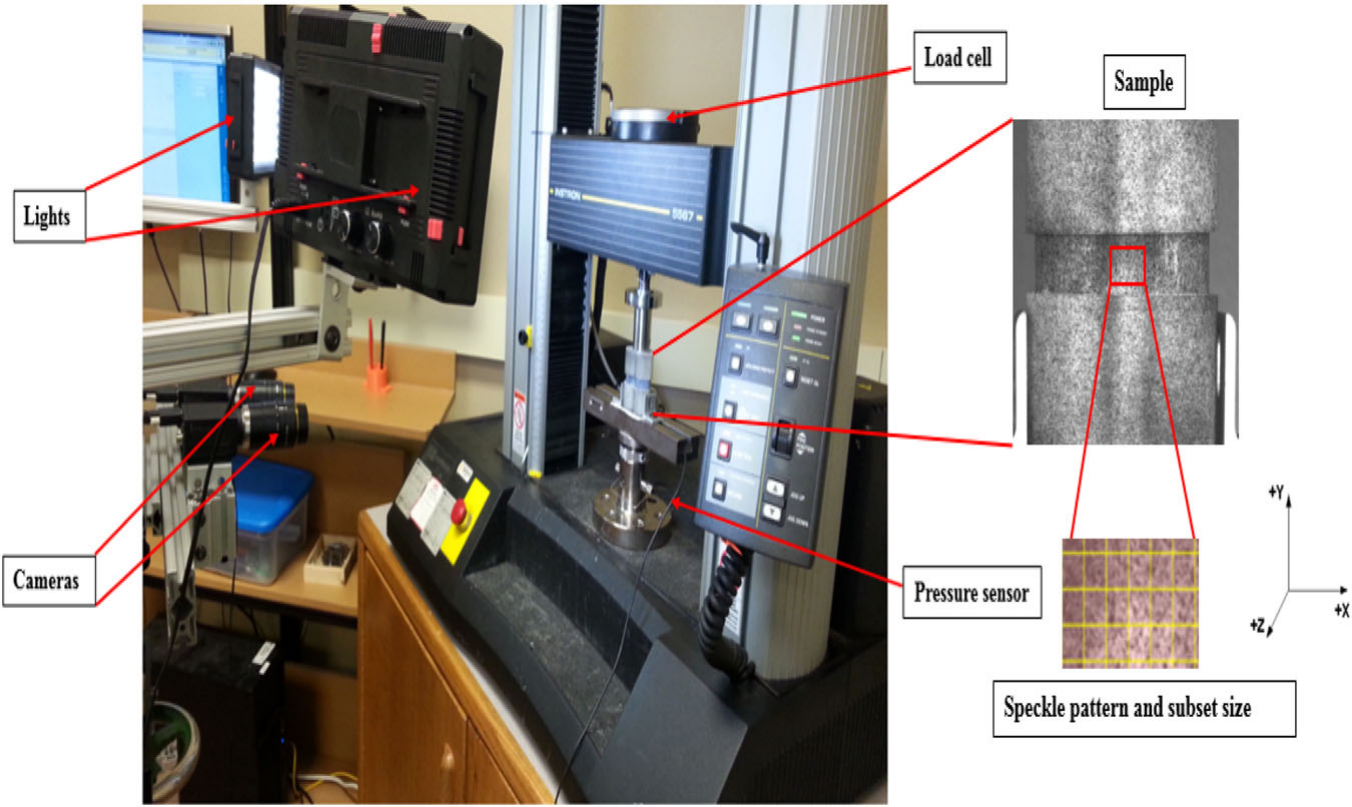
The experimental setup

The device was speckled using standard spray aerosol to create a face contrast that is required for the DIC as shown in Fig. 2. Two cameras were used to capture images of the samples during the compression test. The steps of taking images of the samples began with taking an image of the disc (unreformed) as a reference image. Then, an axial compression load was applied at a rate of 1 mm/min. The images of the disc were captured simultaneously with the known applied load for the deform status for each set of tests, i.e., the disc emulator without NP and with NP as a 4 *wt*. % agarose.

Images were analyzed using Vic-3D software (Correlated Solutions, SC, USA). The subset size of the area of interest was 33 by 33 pixels with 7 pixels spacing to process the images. The 3D Cartesian axis was defined with the *x*-axis representing the displacement in the lateral direction, the *y*-axis in the direction of the applied loads, and the *z*-axis representing the bulging (W) of the disc in the posterior direction. However, only the bulging data (W) will be reported in this study. The bulging measurements were calculated at the mid-height of the disc for each region of interest, i.e., the posterior and posterolateral regions.

The precision of the tests was based on repeated images at zero loads and then calculating the differences between the displacements in three dimensions among these images. The precision of the measurements of the bulging was (0.0030 ± 0.0026 mm).

## Results

**Fig. 3:**
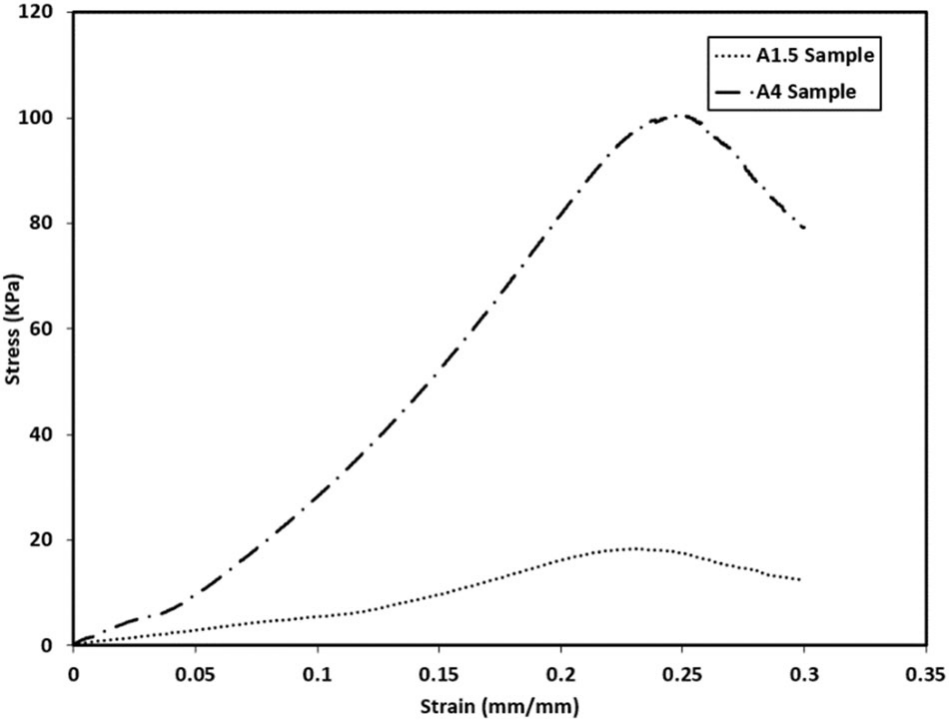
The average stress- strain data of 1.5 *wt*. % agarose and 4 *wt*. % agarose samples under unconfined compression

Stress-strain response of the two hydrogel formulations demonstrated significantly different stiffness and strength as shown in Fig. 3. Stiffness of the 4 *wt*. % solution was ~10× greater than the 1.5 *wt*. % solution, and strength over 5× greater (103.56 + 4.96 kPa vs 18.94 + 1.18 kPa). Both failed at strain levels around 0.25.

**Fig. 4:**
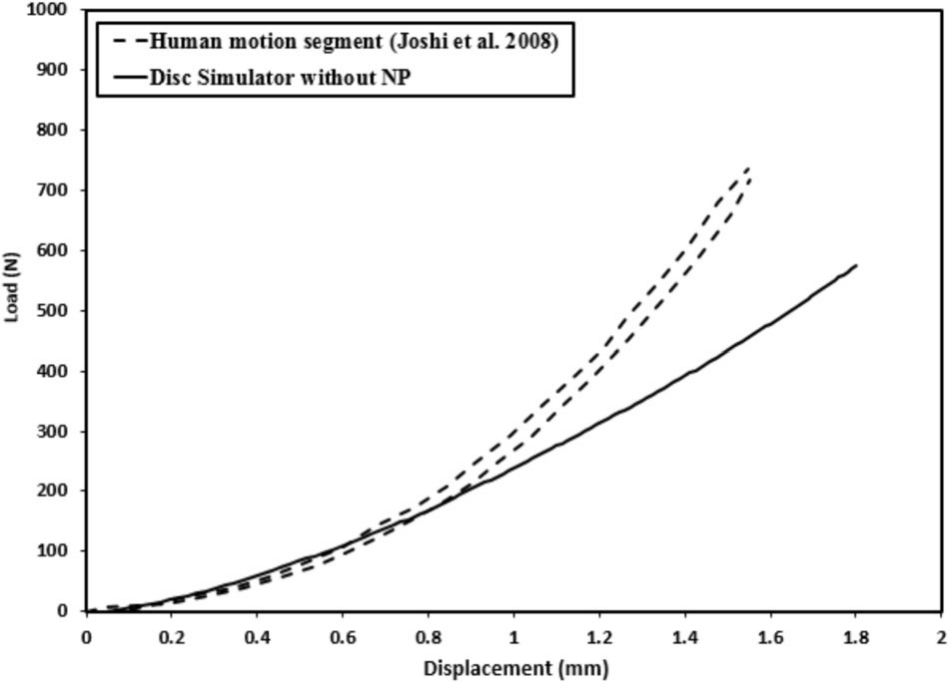
Comparison between the disc emulator data under compression load without NP and reported data of Joshi et al. [4] for the denucleated human motion segment

The disc emulator with an empty nucleus compartment and the 10:1 mixture ratio silicone annulus shows a general agreement with denucleated human lumbar disc response [4] as shown in Fig. 4. Stiffness was very similar to about 1 mm of displacement, then fell behind the stiffening response of the human segment.

**Fig. 5:**
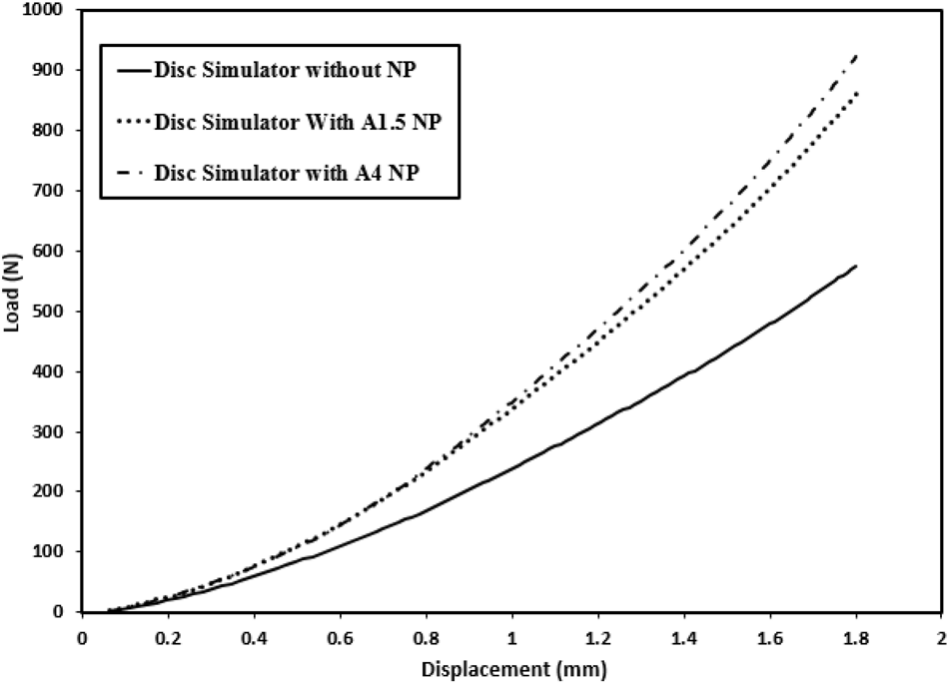
The disc emulator data under compression test without nucleus pulposus NP and with nucleus pulposus, as a reference for comparison (1.5 wt.%, 4 wt.% agarose, A1.5, and A4, respectively)

Adding the NP component to the disc emulator approximately doubled the overall stiffness (Fig. 5) for both the 1.5 *wt*. % and 4 *wt*. % hydrogels. The pressure was maintained during the tests with no leaking of water from the junctions between vertebral bodies and annulus.

**Fig. 6:**
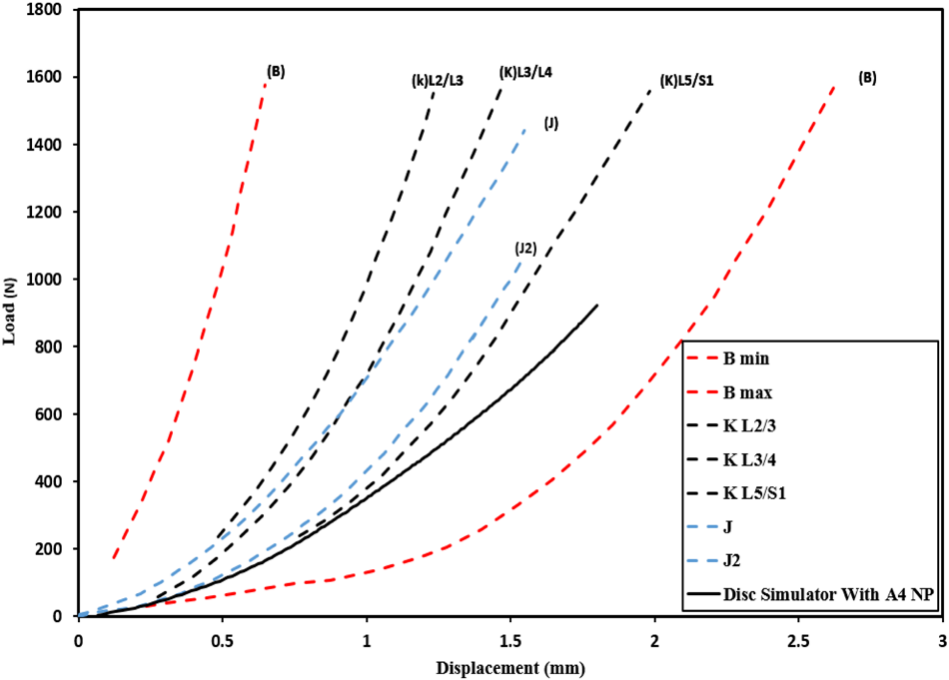
Comparison of the compression response of the disc emulator (4 w/o NP) with literature results: (b) is Brown et al. [3] for segments L2/L3 to L4/L5; (k) is Kulak et al. [7] finite element results; (J) and (J2) are Joshi et al. [4] data for intact human motion segment and implanted hydrogel for NP

Comparisons between the mechanical response of the disc emulator (4 *wt*. % nucleus component) under compression with literature results [3, 4, 7] show good overall agreement (Fig. 6). The initial stiffness of the disc emulator is mid-range, with published test and simulation data showing greater stiffening response as loads increase. Reported data varies widely due to the differences in the biological properties of the various anatomic regions in the spinal column and to the differences in the protocols used [24].

To investigate the bulging direction in the disc, i.e., the artificial annulus fibrosus (AAF) disc, the disc emulator was examined under compression loads to investigate the general trend of the disc emulator in terms of the bulging direction. The disc emulator was used to mimic the human lumbar motion segment under compression loads. The DIC method was also used to study the bulging in the disc. The bulging is defined as the displacement from the non-deformed surface that is perpendicular to the plane at the surface of the assigned region, i.e., the posterior and posterolateral regions. The negative values represent inward bulging, whereas the positive values represent the outward bulging of the disc. Since the posterior element does not have a significant effect on the bulging measurements of the disc [25, 26], the disc emulator was modeled with the posterior element removed.

**Fig. 7:**
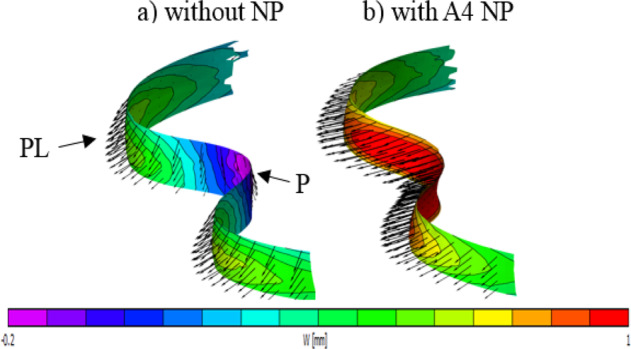
The bulging in the artificial annulus fibrosis disc under a 500 N compressive load, the disc without NP (**a**), the disc with the A4 as an NP (**b**). The big two arrows show the posterior (P) and posterolateral (PL) regions in the disc

Figure 7 shows the typical trend for the bulging map of the surface of the AAF disc in two cases: the NP present, and no NP compartment. This bulging map not only provides a visualization of the bulging in the disc but also enables us to track the bulging direction and magnitude in each region of the disc. The trend of bulging depends on the condition of the NP. It can be seen that the disc bulges outwards when the NP is present. It bulges inwards at the posterior region and outwards at the posterolateral region when the NP is absent.

## Discussion

We developed the disc emulator to add another dimension to spinal disc biomechanics studies. We have shown that a reasonable mechanical analogue of the lumbar spinal motion segment can be generated through simple 3D printing and polymer molding techniques. Overall compression response is similar to the lumbar disc both in intact and denucleated forms, and nucleus pressurization is shown as a dominant stiffening mechanism over inherent hydrogel stiffness.

The disc emulator is unique in that mechanical properties of each component of the disc can be easily and quantifiably altered to match the desired physiological responses of the spinal motion segment, providing a simple and accessible means of testing NP replacement materials, examining the effects of age-related degeneration on AF properties, exploring characteristics of annular defects and mechanisms of nucleus extrusion, and validating finite element models of motion segment behavior.

This initial design is intended to establish basic mechanical characteristics, and there is clearly room for improvement. We observed much less stiffening behavior under increasing loads than the natural system exhibits (Fig. 4), likely due to the monolithic nature of the silicone annulus material. Fiber reinforcement is the basis for stiffening behavior in biological soft tissues, and without it, there is only a small amount of stiffening behavior from large deformation geometric effects. We are pursuing the development of a more complex annulus construct with both the layering and fiber reinforcement seen in the natural system.

The endplates of our device are also a significant simplification, offering some additional compliance and facilitating bonding of the annulus to the vertebral body components, but without porosity. The fluid pumping action across the endplates that porosity engenders is clearly an important phenomenon, and we aim to recapitulate that as well. Pressurization was an initial goal, and that has been demonstrated.

The similar influence of the two hydrogel formulations on construct stiffness, even though the gels themselves varied greatly in unconfined stiffness (Fig. 5), has two important interpretations. First, it is pressurization itself that creates the stiffening effect, and most hydrogels will behave in a similar fashion. Second, simplified tests such as unconfined compression that do not replicate the in situ mechanical conditions of the disc must be used with caution. They alone are unlikely to elicit the key characteristics that will distinguish effective materials for augmentation and replacement from the ineffective.

The outer surface of the annulus is also accessible and applied speckle will support the measurement if disc bulge during testing. Bulging measurements will provide useful comparisons. Therefore, the processed data from the DIC show that the bulging direction of the disc without NP, under axial compression loads, at the posterior region was always inwards, while it bulged outwards when the NP was present (Fig. 7). This trend in the bulging direction agrees with the findings of the available literature [26–28]. For the denucleated disc, the posterior region of the disc deformed inward, which the Seroussi et al. [26] study on human discs showed a similar trend. However, the Meakin et al. [28] study showed that the outer margin of the AF at the posterior region of the sheep disc deformed outwards, while the inner margin deformed inwards.

The bulging at the posterolateral region exhibits higher magnitude outwards four times as much as the posterior region in the denucleated disc, which bulged inwards, under the same value of the compressive loads (Fig. 7a). However, an opposite trend in terms of bulging direction is observed when 4 *wt*.% of agarose was used as an NP, where the posterior region bulged outwards and the bulging is four times greater in magnitude than the posterolateral region under the same magnitude of the compressive loads (Fig. 7b). The outwards bulging in the disc emulator can be interpreted as the NP pressurizes and leads to exert tension forces on the inner wall of the AF. This finding agrees with the Shah et al. [29] study, where they tested human cadaver lumbar disc under compression loads and reported that the maximal radial bulge occurs at the posterolateral region, which is the common region where the prolapse happens. In addition, FE finding by Shirazi et al. [27] predicted the same trend of this study.

This study illustrates that testing the disc emulator under compression loads using the DIC approach has provided qualitative and quantitative bulging measurements. The disc emulator is a reasonable mechanical analogue of the lumbar spinal motion segment and it can be generated by simple 3D printing and polymer molding techniques. The overall bulging response of the disc emulator is like the intact lumbar disc.

## Conclusions

The mechanical compression experiments on the disc emulator confirmed that load-deflection behavior falls within the range of reported values for human tissue samples, in both normal and denucleated conditions. This approach creates a realistic mechanical environment for initial testing of NP replacement materials and evaluating the contributions of individual disc components to overall mechanical behavior. The methodology, based on 3D printing and standard polymer molding methods, is simple, inexpensive, and easy to disseminate among researchers. Improvements in 3D printing can be used within the overall framework to create more realistic simulations and investigate more subtle material and geometric phenomena. Finally, the validation of the response of the disc emulator is expanded by using a DIC approach to quantify disc bulging.
